# Immune factors preceding diagnosis of glioma: a Prostate Lung Colorectal Ovarian Cancer Screening Trial nested case–control study

**DOI:** 10.1093/noajnl/vdz031

**Published:** 2019-09-29

**Authors:** Ivo S Muskens, Mi Zhou, Lucie Mccoy, Paige M Bracci, Helen M Hansen, W James Gauderman, John K Wiencke, Margaret R Wrensch, Joseph L Wiemels

**Affiliations:** 1 Center for Genetic Epidemiology, Department of Preventive Medicine, Keck School of Medicine, University of Southern California, Los Angeles, CA; 2 Department of Epidemiology and Biostatistics; 3 Department of Neurological Surgery, School of Medicine, University of California, San Francisco, San Francisco, CA; 4 Division of Biostatistics, Department of Preventive Medicine, Keck School of Medicine, University of Southern California, Los Angeles, CA

**Keywords:** allergy, glioma, PLCO, TGF-β1

## Abstract

**Background:**

Epidemiological studies of adult glioma have identified genetic and environmental risk factors, but much remains unclear. The aim of the current study was to evaluate anthropometric, disease-related, and prediagnostic immune-related factors for relationship with glioma risk.

**Methods:**

We conducted a nested case–control study among the intervention arm of the Prostate, Lung, Colorectal, and Ovarian Cancer (PLCO) Screening Trial. One hundred and twenty-four glioma cases were identified and each matched to four controls. Baseline characteristics were collected at enrollment and were evaluated for association with glioma status. Serum specimens were collected at yearly intervals and were analyzed for immune-related factors including TGF-β1, TNF-α, total IgE, and allergen-specific IgE. Immune factors were evaluated at baseline in a multivariate conditional logistic regression model, along with one additional model that incorporated the latest available measurement.

**Results:**

A family history of glioma among first-degree relatives was associated with increased glioma risk (OR = 4.41, *P* = .002). In multivariate modeling of immune factors at baseline, increased respiratory allergen-specific IgE was inversely associated with glioma risk (OR for allergen-specific IgE > 0.35 PAU/L: 0.59, *P* = .03). A logistic regression model that incorporated the latest available measurements found a similar association for allergen-specific IgE (*P* = .005) and showed that elevated TGF-β1 was associated with increased glioma risk (*P*-value for trend <.0001).

**Conclusion:**

The results from this prospective prediagnostic study suggest that several immune-related factors are associated with glioma risk. The association observed for TGF-β1 when sampling closer to the time of diagnosis may reflect the nascent brain tumor’s feedback on immune function.

**Importance of the Study**

This nested case–control study within the intervention arm of the Prostate, Lung, Colorectal, and Ovarian Cancer (PLCO) Screening Trial replicated that allergen-specific IgE is inversely associated with glioma risk. This is also the first prospective study to show that a family history of glioma is associated with increased glioma risk, which is consistent with the literature. TGF-β1, which was analyzed in prospectively collected serum samples, was not associated with glioma risk at baseline but became significant closer towards diagnosis, which may reflect the presence of a tumor before tumor diagnosis or manipulation of the immune system by the tumor prior to diagnosis. These results are indicative of a protracted prodromal immune impact of glioma which may be capitalized for early detection, prevention, or treatment modalities to improve outcomes for glioma patients.

**Key Points**

- 1. This study replicated that allergen-specific IgE is inversely associated with glioma risk.
- 2. A family history of glioma among first-degree relatives is associated with glioma.
- 3. TGF-β1 became associated with glioma closer towards diagnosis.

Over recent decades, substantial progress has been made with regard to the understanding of glioma epidemiology.^1^ A long-established risk factor for glioma is a history of exposure to ionizing radiation.^1,2^ Several recent studies have identified common germline genetic variants that are associated with increased risk.^3^ Various other factors including family history and allergy or atopy have also been consistently associated with glioma risk.^4–8^

Apart from the question of glioma risk factors, another relevant question remains whether certain factors may reflect an underlying disease process before glioma diagnosis. Although the lifetime history of immune-related diseases such as allergy and autoimmunity seems to influence gliomagenesis,^9,10^ the tumor itself clearly exhibits immunoevasive features such as secretion of immunomodulatory cytokines, altered expression of immunoregulatory receptors, and manipulation of the immune cellular landscape proximal to the tumor.^11^ One of the most clinically relevant questions is how long a glioma may reside within the brain before it becomes symptomatic, and if and how it may undergo malignant transformation to higher grade gliomas. This is likely to vary by the type of glioma. We believe that part of this process will be found to include immunomodulatory manipulations.

This study was designed to evaluate characteristics at study entry and immune factors within prediagnostic blood samples from the Prostate Lung Colorectal Ovarian Cancer (PLCO) Cancer Screening Trial among glioma cases and matched controls.^12,13^ The original aim of the PLCO Cancer Screening Trial was to evaluate screening methods in stored blood samples for utility in screening for common cancers.^12,13^ The PLCO Cancer Screening Trial was extended to include other diseases identified during follow-up,^12,13^ allowing for evaluation of various immune factors and their association with glioma risk at baseline and over time.

## Study Population and Methods

### Study Population 

The PLCO Cancer Screening Trial was a prospective randomized cancer screening trial with the aim of evaluating the utility of X-rays for lung cancer diagnosis, sigmoidoscopy for colorectal cancer, CA125 for ovarian cancer, and Prostate Specific Antigen (PSA) for prostate cancer as screening tools (NCT00339495). A total of 154,897 subjects between ages 55 and 74 were randomized between 1993 and 2001 at 10 centers located across the United States (Alabama, Colorado, Hawaii, Michigan, Minnesota, Missouri, Pennsylvania, Utah, Washington D.C., and Wisconsin). All participants provided informed consent and completed a self-administered baseline questionnaire.^14^ All participants were cancer-free at inclusion and received routine care with those in the intervention arm also received cancer screening. Participants in the intervention arm also provided blood samples at five predefined times during the trial and form the basis of the Etiology and Early Markers Study (EEMS), an ongoing observational cohort with prediagnostic specimens. Follow-up for all participants lasted until at least 2010. Cancer occurrences were ascertained by annual mailed questionnaire (>95% follow-up rate) and verified through death certificates and medical records. Ethical approval was obtained at all PLCO Cancer Screening Trial sites. The current study was performed on de-identified samples and data from the PLCO Cancer Screening Trial, and was approved by IRBs at the University of California San Francisco (UCSF), University of Southern California (USC), and the National Cancer Institute Special Studies Institutional Review Board (NCI-SSIRB).

### Nested Case–Control Study 

Only participants in the PLCO intervention arm were used for this report because of the availability of blood samples. During the follow-up period, newly diagnosed glioma cases were identified as those diagnosed with International Classification of Diseases for Oncology version 2 (ICD-O-2) site codes: C71.0-C71.9. All cases had a histopathological glioma confirmation and the glioma diagnosis was the first cancer diagnosis for each individual glioma case. A total of 124 glioma cases were identified and matched to 496 cancer-free controls (1:4 ratio) based on age at enrollment (<60, 60–64, 65–69, ≥70), sex, ethnicity, and month of blood draw (2 month groups starting at January/February, March/April, etc.).

### Exposure Assessment

Demographics, such as weight, height, smoking history, cancer history among first-degree relatives, glioma history among first-degree relatives, diabetes, hypertension, comorbidities, and other potential risk factors for glioma, were obtained from the baseline questionnaire administered at study entry. Data on exogenous hormone use, parity, age of the first child, and menopausal status were collected for female participants. Prostate-related factors were also evaluated among male participants. Serum samples for subjects in our nested case–control study were transferred to University of California at San Francisco for conducting immune-related measurements, including TGF-β1, TNF-α, total IgE, and respiratory allergen-specific IgE (Phadiatop: mite, oak, ragweed, grass, dog, cat, and Alternaria).^15^ Immune factors were measured using serum samples collected at baseline for all cases and controls. Additional repeated serum samples were obtained by PLCO staff at years 1, 2, 4, and 5 of follow-up. However, only a subset of the repeated serum samples was analyzed, with a higher proportion being requested from cases. One or more repeated (post-baseline) serum samples were obtained for 87% of cases (*n* = 108) and 15% of controls (*n* = 72). Immune factors were measured using Luminex for the cytokines (Millipore) and Phadia for the two IgE-related measures. All tests were performed in duplicate and in reference to appropriate standard curves. When duplicate measurements had a coefficient of variation of over 20%, the test was redone. Duplicate measures were averaged for analysis.

### Statistical Analysis

Height and weight at age 20 and at study entry were determined, and each was categorized based on quartile cutpoints in controls for use in statistical models. Body mass index (BMI) at age 20 and study entry was calculated as weight in kg divided by height in m^2^ and categorized as <25, 25 to <30, and ≥30 kg/m^2^. Smoking status was categorized as never smoker, past smoker, and current smoker. IgEs were classified according to clinically accepted criteria and were categorized as elevated if >100 kU/mL.^16^ Respiratory allergen-specific IgE was categorized as elevated if >0.35 PAU/L.^10,17^ TGF-β1 was categorized by quartiles in controls at baseline (with cutpoints 1,027, 1,284.6, and 1,533.2 pg/mL). TNF-α was categorized by quartiles in controls at baseline (with cutpoints 12.4, 16.5, and 22.0 pg/mL). Variables that were evaluated by quartiles were also visualized using boxplots by case–control status and by measurement year and were created in R (version 3.6.0).

The association between evaluated baseline characteristics and glioma was estimated by odds ratios (ORs) and 95% confidence intervals (CIs) using conditional logistic regression. All models for BMI and weight were adjusted for diabetes status and education. Models for smoking history were adjusted for diabetes status, education, and hypertension, and models for a family history of cancer or glioma were adjusted for smoking status. BMI, weight, and height were analyzed as categorical variables in all models. *P*-values for trend were calculated for BMI, weight, height, and factors that were categorized by quartiles by entering the ordinal values representing categories for these factors as continuous variables in the models to evaluate trends. Conditional logistic regression was performed using the “clogistic” function from the Epi package in R.^18,19^ Immune factors were evaluated using a multivariate conditional logistic regression model with correction for batch number. An additional multivariate logistic regression model was created, which incorporated measurements from the latest available serum sample when available. This model was adjusted for age, sex, ethnicity, and month of blood draw as matching had to be broken for this analysis.

## Results

The baseline characteristics of glioma cases and controls are depicted in Table 1. The mean age at entry was 63 years (SD = 5.2). The majority of both cases and controls were male (65%). Non-Hispanic whites were the most common ethnicity (95%), followed by non-Hispanic blacks (2%), and Hispanics (1%). Observation time for cases ended at diagnosis and therefore cases (mean 6.5 years) were observed for a shorter time than controls (mean 12.4 years, *P* < .001). Ninety-three out of the 124 glioma cases were diagnosed with a glioblastoma (GBM, 75.0%).

**Table 1.** Baseline characteristics for the glioma cases and controls for matching variables and days to case–control status

Variable	Cases (*N* = 124)	Controls (*N* = 496)	*P*-value	Overall (*N* = 620)
**Sex (*N* (%))**				
** Male**	81 (65)	324 (65)	>.99	405 (65)
** Female**	43 (35)	172 (35)		215 (35)
**Ethnicity (*N* (%))**				
** Non-Hispanic White**	118 (95)	472 (95)	>.99	590 (95)
** Hispanic**	1 (1)	4 (1)		5 (1)
** Black non-Hispanic**	3 (2)	12 (2)		15 (2)
** Other**	2 (2)	8 (2)		10 (2)
**Age (years (SD)**				
** Mean (SD)**	63.5 (±5.0)	63.4 (±5.3)	.884	63 (5.2)
**Time to disease (cases) or censoring (controls, in years (SD))**				
** Mean (SD)**	6.5 (3.9)	12.4 (2.6)	<.001	11.2 (3.7)

The table depicts the variables that were used to match cases to controls. *P*-values were calculated using t-test for continuous variables and chi-squared test for categorical variables.

A family history among first-degree relatives of glioma was associated with increased glioma risk (OR = 4.41, *P* = .002, Table 2). A history of any cancer among first-degree relatives was not associated with increased glioma risk (*P* = .18). Increased weight (Q3 with Q1 as reference) was associated with increased glioma risk (*P* = .03), but the pattern across quartiles was not consistent (trend *P*-value = .12). No other anthropometric factor at baseline was significantly associated with case–control status. Smoking status was not associated with glioma risk. No comorbidity evaluated at baseline was associated with glioma risk (Supplementary Table S1). None of the evaluated factors specific to males and females were associated with glioma risk (Supplementary Tables S2 and S3).

**Table 2.** Associations between anthropometric factors, smoking history, and family history of cancer and glioma among first-degree relatives at baseline

Variable	Cases	Controls	OR	95% CI		*P*-value	*P*-value for trend
				Low	High		
**BMI (kg/m** ^**2**^)^**a,b**^							
** <25**	40	151	Ref.				.86
** 25 to <30**	53	226	1.01	0.59	1.73	.97	
** ≥ 30**	29	115	1.06	0.57	1.98	.86	
**BMI at age 20 (kg/m** ^**2**^)^**a**^							
** <25**	93	419	Ref.				.15
** 25 to <30**	23	62	1.74	0.93	3.24	.08	
** ≥30**	3	9	1.10	0.20	6.02	.91	
**Weight (kg)** ^**a**^							
** Q1 (<70.6)**	26	124	Ref.				.12
** Q2 (≥70.6 to <79.8)**	24	123	1.13	0.51	2.48	.76	
** Q3 (≥79.8 to <90.7)**	48	133	2.26	1.09	4.69	**.03**	
** Q4 (≥90.7)**	25	116	1.49	0.66	3.35	.34	
**Height (cm)**							
** Q1 (<165.2)**	30	140	Ref.				.09
** Q2 (≥165.2 to <175)**	34	125	1.49	0.78	2.84	.22	
** Q3 (≥175 to <182)**	26	135	1.19	0.51	2.74	.69	
** Q4 (≥182)**	32	92	2.16	0.93	5.02	.07	
**Smoking history** ^**c**^							
**Nonsmoker**	54	222	Ref.				
** Past smoker**	61	240	1.04	0.64	1.68	.88	
** Current smoker**	9	34	1.89	0.71	5.01	.20	
**Family history of cancer** ^**d**^							
** Family history of cancer**	77	275	1.33	0.88	2.00	.18	
**Family history of glioma** ^**d**^							
** Family history of glioma**	9	8	4.41	1.69	11.47	**.002**	

Height and weight were evaluated by quartiles among controls.

^a^BMI, BMI at age 20, and weight were adjusted for diabetes status and education.

^b^Current BMI.

^c^Smoking history was adjusted for diabetes status, education, and hypertension.

^d^Family history of cancer and glioma were adjusted for smoking status.

Bold values indicate significant P-values (i.e., P < .05).

**Fig. 1:**
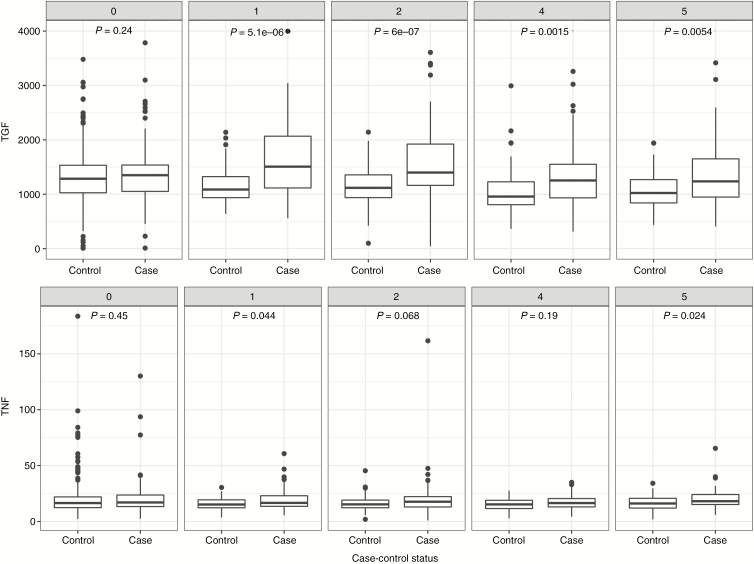
Boxplots depicting TGF-β1 and TNF-α by case-control status and by measurement year. The boxplots depict the measures of TGF-β1 and TNF-α by case–control status for each of the years of measurements (0, 1, 2, 4, and 5). No measurements were taken at year 3. *P*-values were derived from *t*-tests.

Comparison of the distribution of TGF-β1 and TNF-α appears to indicate that, at later years of measurement, higher TGF-β1 is observed among cases compared with controls (Figure 1). In multivariate modeling of immune factors at baseline, increased respiratory allergen-specific IgE was inversely associated with glioma risk (OR for IgE > 0.35 PAU/L: 0.59, *P* = 0.03, Table 3). No significant associations were observed for TGF-β1, TNF-α, and total IgE. The multivariate logistic regression model that incorporated the latest available measures also showed that increased respiratory allergen-specific IgE was significantly inversely associated with glioma risk (OR for respiratory allergen-specific IgE > 0.35 PAU/L: 0.48, *P* = .005, Table 4). Unlike the baseline model, increased TFG-β1 was associated with increased glioma risk (OR for TGF-β1: Q4 vs. Q1: 2.96, *P*-value for trend: <.0001, Table 4).

**Table 3.** Multivariate conditional logistic regression model for immune factors at baseline for association with glioma

	OR	95% CI		*P*-value	*P*-value for trend
		Low	High		
**Total IgE (kU/mL)**					
** Normal (≤100)**	Ref.				
** Elevated (>100)**	1.06	0.62	1.83	.82	
**Respiratory allergen-specific IgE (PAU/L)**					
** Normal (≤0.35)**	Ref.				
** Elevated (>0.35)**	0.59	0.37	0.95	**.03**	
**TNF-α (pg/mL)**					.56
** Q1 (≤12.4)**	Ref.				
** Q2 (>12.4 to ≤16.5)**	0.89	0.48	1.65	.72	
** Q3 (>16.5 to ≤22.0)**	0.95	0.52	1.73	.85	
** Q4 (>22.0)**	1.18	0.65	2.15	.59	
**TGF-β1 (pg/mL)**					.14
** Q1 (≤1,027)**	Ref.				
** Q2 (>1,027 to ≤1,284.61)**	0.98	0.52	1.85	.95	
** Q3 (>1284.61 to ≤1,533.15)**	1.60	0.90	2.85	.11	
** Q4 (>1,533.15)**	1.36	0.73	2.52	.34	

ORs were stratified by matching set and adjusted for batch number.

OR = odds ratio; CI = confidence interval.

Bold values indicate significant P-values (i.e., P < .05).

**Table 4.** Multivariate logistic regression model using the latest available measurements for each study participant

Variable	OR	95% CI		*P*-value	*P*-value for trend
		Low	High		
Total IgE (kU/mL)					
Normal (≤100)	Ref.				
Elevated (>100)	0.88	0.48	1.55	.67	
Respiratory allergen-specific IgE (PAU/L)					
Normal (≤0.35)	Ref.				
Elevated (>0.35)	0.48	0.28	0.97	**.005**	
TNF-α (pg/mL)					.60
Q1 (≤12.4)	Ref.				
Q2 (>12.4 to ≤16.5)	1.05	0.56	2.00	.87	
Q3 (>16.5 to ≤22.0)	1.40	0.76	2.59	.28	
Q4 (>22.0)	1.07	0.58	2.01	.82	
TGF-β1 (pg/mL)					**<.0001**
Q1 (≤1,027)	Ref.				
Q2 (>1,027 to ≤1,284.61)	0.80	0.40	1.55	.50	
Q3 (>1,284.61 to ≤1,533.15)	1.43	0.76	2.69	.27	
Q4 (>1,533.15)	2.96	1.68	5.31	**.0002**	

ORs were stratified by matching set and adjusted for batch number.

OR = odds ratio; CI = confidence interval.

Bold values indicate significant P-values (i.e., P < .05).

## Discussion

In this study, glioma cases and matched controls among the intervention arm of the EEMS component of the PLCO Cancer Screening Trial were evaluated for baseline characteristics and various immune-related factors in prediagnostic blood serum for association with glioma case–control status. With regard to baseline characteristics, this study supported a role for family history of glioma among first-degree relatives and increased glioma risk but did not demonstrate significant associations with various anthropometric factors such as BMI and height. We also evaluated various immune factors for association with glioma case–control status and showed that elevated respiratory allergen-specific IgE was associated with decreased risk. This association was also identified in a logistic regression model that incorporated the latest available measurements to account for updated longitudinal measurements in a portion of the study population. The logistic regression that incorporated the latest available measurements showed that elevated TGF-β1 is associated with increased glioma risk.

A family history of glioma among first-degree relatives has previously been associated with increased glioma risk,^5–7,20^ which is consistent with our study. To the best of our knowledge, this is the first prospective cohort study to show that a family history of glioma is associated with increased risk. The result is also unlikely to be affected by diagnostic bias, since this history was collected prior to glioma diagnosis among study subjects. This finding may be explained by families harboring rare putative germline mutations that result in increased risk, which is supported by studies that identified rare germline variants in glioma predisposing genes among such families.^21–23^ It has even been suggested that family members of a particular family with multiple reported gliomas tend to develop gliomas of the same molecular subtype.^24^ Increased height has previously been associated with increased glioma risk,^25,26^ but this was not observed in this study.

The negative association between allergen-specific IgE and glioma risk has been previously identified,^10^ but this was not significant in another study.^27^ A reported history of atopy has also previously been associated with decreased glioma risk.^4,8^ However, a recent Mendelian randomization study only provided weak evidence for an association between atopy risk and glioma^28^; hence, the genetic component of this risk may be low. Higher prediagnostic total IgE has also been associated with decreased glioma risk,^10^ although one other study did not observe this association.^27^ A meta-analysis using a combination of studies of pre- and post-diagnostic sera identified that higher total IgE was associated with decreased risk, but that respiratory allergen-specific IgE was not,^29^ which is not in line with the findings of the current study as total IgE was not associated with glioma case–control status. We were not able to assess self-reported allergy as the PLCO cohort had not collected data on this factor, but our result is in line with other studies that assessed hay fever and other reported measures of respiratory allergy as being inversely associated with glioma.^9,30^

To the best of our knowledge, this is the first study to report an association between prediagnostic serum TGF-β1 with regard to glioma risk. Our findings indicate that increased prediagnostic TGF-β1 may reflect an underlying disease process but may also be produced by the systemic immune system in response to the glioma. Inhibition of TGF-β has been shown to improve glioma survival in a mouse model potentially due to inhibition of tumor filtration and restoration of the immune surveillance.^31^ TGF-β1 greatly influences the immune system through influencing T-cell development, limiting B-cell proliferation and differentiation, limiting the development of natural killer cells, and limiting dendritic cell functioning.^32–34^ It has been suggested that this immunologic inhibition by TGF-β1 is necessary for the creation of tolerance for self- and innocuous antigens such as gut bacteria and food and TGF-β1 malfunctioning may result in severe autoimmunity.^34^ Secretion of TGF-β1 by glioma cells may, therefore, facilitate an immunotolerant environment necessary for cancerous growth. TGF-β may result in T-cell sequestration in the bone marrow among GBM patients through down-regulation of S1P1,^35^ but it should be noted that TGF-β blockade did not result in less T-cell sequestration.^35^ Glioma cell line studies have also shown that glioma cells produce TGF-β1 for retention of stemness of glioma initiating cells (GICs) through an induced expression of various genes including *Sox2*, a stemness gene.^36,37^ Furthermore, TGF-β1 has been shown to promote cell migration and invasiveness in glioma and may be involved in angiogenesis.^38–40^

Although these results have not yet been validated by other studies, they may provide a potential roadmap for early detection and treatment of prodromal glioma prior to clinical symptoms. TGF-β1 and other factors such as tumor-secreted exosomes^41^ and germline mutations^3^ may be used clinically to identify people at high risk for glioma, identify cases before symptoms occur, and identify patients with tumors responsive to anti-TGF-β therapy. While targeting the TGF beta pathway is under development and has been problematic,^42,43^ other prediagnostic markers certainly need to be discovered for the use of high-quality prediagnostic cohorts may help facilitate developments in preclinical prevention of a deadly disease that has resisted clinical breakthroughs for decades.

## Strengths and Limitations

This study has various strengths and limitations. The study’s primary strengths are the availability of prediagnostic serum samples, prospective nature, relatively large sample size, and long duration of follow-up. Although the longitudinal samples are helpful, one of the limitations is incompleteness of analyzed repeated serum samples in the logistic regression model that incorporated the latest available measurements which were the result of selection considerations. Estimates may, therefore, be less precise because of the incompleteness of the follow-up measures. The use of completely cancer-free controls may also have been too restrictive. Cases were relatively oversampled for repeated measures compared with controls, but we believe that this has not introduced bias as the selection of cases and controls and the selection of serum samples was not based on any criteria other than availability for cases. At 75%, GBM was a more common diagnosis than would be expected in the general population^44^ probably due to the age of inclusion of the PCLO Cancer Screening Trial. Another limitation is that TGF-β1 was the only TGF isoform to be evaluated.

In conclusion, this study suggests that certain lifetime immune-related factors may be associated with glioma risk long before diagnosis such as respiratory allergen-specific IgE. This study also shows that TGF-β1 becomes associated with glioma risk closer towards diagnosis which may reflect the underlying disease process. Further evaluation in other and larger glioma cohorts with access to prediagnostic blood samples are needed to confirm these associations and to evaluate potential clinical utility as a diagnostic or screening tool.

## Funding 

This study was supported by U01CA182371 from the NIH.

## Supplementary Material

vdz031_suppl_Supplementary_Tables

## Notes

**Authorship statement:** Concept of study: JLW, laboratory work and analyses: MZ, LM, HH, PMB, statistical analyses: ISM, WJG, JLW, draft of the manuscript: ISM, JLW, careful review of manuscript: MZ, LM, PMB, HH, JKW, MRW, WJG.
